# Notices and Reviews

**Published:** 1890-03

**Authors:** 


### Book Notices.

THE PHYSICIAN HIMSELF AND THINGS THAT CONCERN HIS REPUTATION AND SUCCESS. By D. W. Cathell, M. D., Baltimore, Maryland. Ninth Edition. Revised and enlarged. F. A. Davis, publisher, Philadelphia and London, 1889.

This work teaches professional tact and business sagacity. It elaborates a great deal of the Code of Ethics, both written and unwritten, and emphasizes those things concerning which no well regulated physician can afford to ignore. It teaches how to succeed, how to make a reputation and how to be a gentleman.

In answering the question, "How shall I conduct myself in the profession, and what honorable means can I employ in addition to scientific knowlege and book-learning, in order to make my success more certain, more rapid and more complete, and to excel in the great battle of life to the fullest extent that lies in me?" the author says, "First, last, and in the midst of all, you should, as a man and as a physician, always, and above all else, keep whatever is honest, whatever is true, whatever is just, and whatever is pure foremost in your mind, and be governed by it.

"If you do not you ought not to succeed in the profession, and no honest man can wish you success."

A great deal of the chapter on religion could well be eliminated.

The exposition of sectarianism in medicine, including all kinds of pathies, is the best the writer has ever read.

The author defines rational medicine to mean everything that is good for sick humanity, regardless of its source. This is the science of medicine, the regular profession, and is as broad as the universe. B.

ELECTRICITY IN THE DISEASES OF WOMEN, with special reference to the application of strong currents. By G. Belton Massey, M. D. Philadelphia: F. A. Davis, 1889.

This little volumn contains 200 pages, bound in cloth. It speaks very frankly about electricity in diseases of women and makes a great many statements and provisos. Any one interested in the subject will find food for thought in the book. B.

---

DIPHTHERIA, its Nature and Treatment, by C. E. Billington, M. D., and Intubation in Croup, and other Acute and Chronic Forms of Stenosis of the Larynx, by Joseph O'Dwyer, M. D. Octavo, 326 pages. Price, muslin, $2.50. New York, William Wood & Co.

Dr. Billington's book is just from the press and is replete with all that is new and interesting in the way of etiology, pathology and therapeutics of diphtheria. The subject matter is concise and well arranged. Evidently the author is a practical physician, and no doubt his work will meet with a large sale.

Dr. Joseph O'Dwyer has occupied the last 40 or 50 pages of the book with a complete explanation and treaties on intubation. This method seems to be on the wane, but the father of the procedure in this country is Dr. O'Dwyer, and anyone wishing to be authoritatively informed on the subject would do well to study this book. B.

---

AMERICAN RESORTS, with Notes upon their Climates. By Bushood W. James, A. M., M. D. Philadelphia: F. A. Davis, 1889.

This work contains 270 pages, coarse print, and a comprehensive map of the United States, Canada and Mexico, gotten up especially for the book. From what the writer personally knows of the countries and resorts described in the book, he is satisfied that the truth prevails throughout its pages. This is saying a great deal for a work of this kind. It is the custom to exaggerate on countries and climates, but Dr. James' book is to be commended for fairness of statements. Texas, and especially Austin, is mentioned very favorably as a health resort. B.

---

IMMUNITY through Leucomaines. By Eusebio Guell Bacigalupi; translated from the Second French edition by R. F. Rafael, M. D. New York: J. H. Vail & Co., 1889.

This little book is very interesting and ingenious. Paper cover, 170 pages. It explains vaccination, how it produces immunity in certain cases, etc. It is well worth any one's time to read through the pages of this book.

---

LECTURES ON NERVOUS DISEASES from the standpoint of cerebral and spinal location, and the later methods employed in the diagnosis and treatment of these affections. By Ambrose L. Ranney, A. M., M.|D. Professor of the Anatomy and Physiology of the Nervous System in the N. Y. Post-Graduate Medical School and Hospital. Profusely illustrated with original diagrams and sketches in color by the author; carefully selected wood cuts, and reproduced photographs of typical cases. Philidelphia: F. A. Davis, Publisher.

This book brings the subject of cerebral and spinal localization well up to the present time, and presents the subject in a very readable and attractive style. Professor Ranney has been a teacher a long time, and his large experience is quite manifest in the work. With all the good points and great value of the book as a whole, the reviewer is inclined to look upon the section devoted to "eye-defect" and "eye-strain" as a trifle sensational. B.

---

THE URINE, the Common Poisons and the Milk. Memoranda, chemical and microscopical, for laboratory use. By J. W. Holland, M. D., Professor of Medical Chemistry and Texicology, Jefferson Medical College of Philadelphia. Illustrated; third edition, revised and much enlarged. P. Blockiston, Son & Co., Philadelphia. B.

---

CHAMBERS' Visiting List for 1890 is received. Price, net, 75c. A very handy and good think for the doctor's daily use.

